# Association between cigarette smoking and hearing loss: A cross-sectional study from the NHANES database

**DOI:** 10.18332/tid/208812

**Published:** 2025-09-17

**Authors:** Hesen Huang, Wenkao Zhou, Kaiqin Chen, Yu Du, Wei Lin, Yixian Ye

**Affiliations:** 1 Department of Otolaryngology, The Seventh Affiliated Hospital, Sun Yat-sen University, Shenzhen, China; 2 Department of Emergency Medicine, Xiang'an Hospital, Xiamen University, Xiamen, China; 3 Department of Neurosurgery, Xiang'an Hospital, Xiamen University, Xiamen, China; 4 Department of Otolaryngology-Head and Neck Surgery, Xiang'an Hospital, Xiamen University, Xiamen, China; 5 Department of Otolaryngology-Head and Neck Surgery, National Regional Medical Center, The First Affiliated Hospital, Fujian Medical University, FuZhou, China; 6 Department of Otolaryngology, Zhongshan Hospital, Fudan University, Xiamen, China

**Keywords:** smoking, hearing loss, NHANES database, cross-sectional study, association

## Abstract

**INTRODUCTION:**

Using data from the National Health and Nutrition Examination Survey (NHANES), this study aimed to assess the association between smoking and hearing loss and explore its heterogeneity across gender and age groups.

**METHODS:**

This secondary dataset analysis used a cross-sectional design and included NHANES data from 2005–2012 and 2015–2018. The study population consisted of adults without hearing-related disorders. Hearing loss was assessed by pure tone audiometry (PTA) and included low-frequency (LFHL), speech-frequency (SFHL) and high-frequency hearing loss (HFHL), expressed as continuous and categorical variables, respectively. Linear and logistic regression models were used to analyze the association between hearing loss and the exposure variable smoking status.

**RESULTS:**

A total of 4217 adult subjects were included. It was found that smokers had a higher prevalence of LFHL, SFHL and HFHL than non-smokers (LFHL: 15.62% vs 8.51%, SFHL: 23.22% vs 12.98%, HFHL: 53.48% vs 36.95%). In males, in Models 1 (unadjusted), 2 and 3 (adjusted), there were statistically significant differences (p<0.05) in LFHL (β=4.24; 95% CI: 3.32–5.17; β=1.65; 95% CI: 0.80–2.49; β=1.52; 95% CI: 0.66–2.38) in SFHL (β=5.63; 95% CI: 4.56–6.70; β=1.95; 95% CI: 1.05–2.84; β=1.62; 95% CI: 0.72–2.52) and in HFHL (β=10.20; 95% CI: 8.21–12.19; β=2.85; 95% CI: 1.33–4.37; β=2.19; 95% CI: 0.69–3.70) between smokers and non-smokers, for continuous variables of hearing loss. In male hearing loss, categorical variables also showed statistically significant differences between smokers and non-smokers (p<0.05). In the middle-aged group, compared with non-smokers, logistic regression of smoking with all three types of hearing loss showed statistically significant differences (p<0.05) in Models 1, 2 and 3.

**CONCLUSIONS:**

There was a significant association between smoking and hearing loss, with maybe more significant associations with all three types of hearing loss in male smokers and a significant relationship between smoking and hearing loss in the middle-aged group.

## INTRODUCTION

According to the World Health Organization (WHO), 466 million people worldwide have disabling hearing loss, and it is expected that by 2050, more than 900 million people will have hearing loss^1^. Hearing loss impairs the ability to understand speech, makes communication and social contacts difficult, and is a burden on families and society, costing the world economy US$750 billion a year. It has become the second most common non-fatal problem affecting human health^2^. Hearing loss is caused by pathological conditions of the auditory pathway, which has multiple risk factors: genetic causes, birth complications, infectious diseases, chronic ear infections, use of ototoxic drugs, exposure to noise, sex, and aging^3-5^. Several studies have also shown that hearing loss is also associated with hypertension, lethargy, and smoking^6-9^.

Tobacco smoking is the most common global public health problem and is recognized as a potential risk factor for the most life-threatening chronic diseases that can lead to harm, such as cancer (lung, throat, blood, etc.), cardiovascular, and respiratory diseases^10,11^. In addition to systemic diseases caused by tobacco, studies have found that smoking also affects the senses, which include hearing^9^. Compared to non-smokers, smokers are at a higher risk of hearing loss, the hearing system is greatly affected by smoking, and a biomarker of tobacco exposure in active and passive smokers is cotinine (an alkaloid found in tobacco that is a major metabolite of nicotine)^2,6,7^. Patel et al.^12^ found that smoking was associated with recurrent acute otitis media, exudative otitis media and sensorineural hearing loss in children exposed to secondhand smoke. In adults, it was related to active and aggressive chronic suppurative otitis media, poorer success of tympanoplasty, increased postoperative complications, and sensorineural hearing loss with more pronounced high-frequency decline. Similar studies have previously shown an association between smoking and hearing loss^2,6,13,14^, but others have reported no association between smoking and hearing loss^15,16^.

As the relationship between smoking and hearing loss remains controversial, we utilized data from the National Health and Nutrition Examination Survey (NHANES) to determine the effects of smoking on hearing impairment in the general population, and further explored the effects of smoking on hearing in different gender and age subgroups.

## METHODS

### Study population and study design

**Figure 1:**
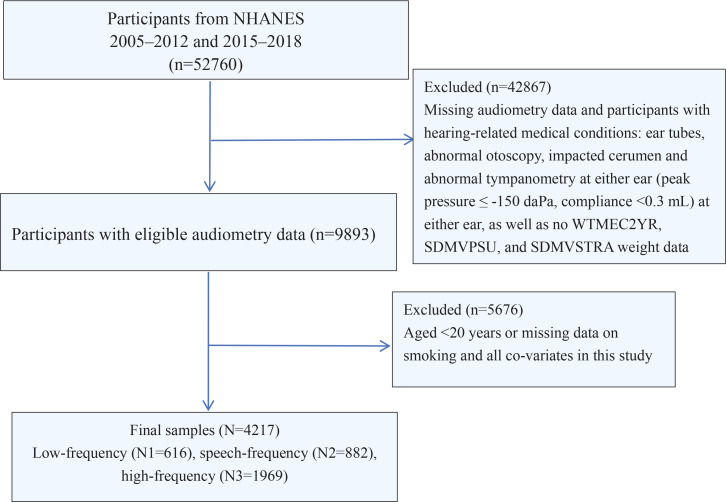
The screening process flowchart for participants

This is a secondary dataset analysis of the NHANES dataset. All participant data for this cross-sectional study were obtained from the NHANES database (2005–2012 and 2015–2018), a research project that assesses the health and nutritional status of civilian and non-institutionalized populations in the US. NHANES is multi-purpose research project conducted by the National Center for Health Statistics (NCHS) and the Centers for Disease Control and Prevention (CDC)^17^. Information data are collected through a combination of questionnaires, physical examinations, and laboratory tests^18^. More information is available at https://wwwn.cdc.gov/nchs/nhanes/Default.aspx. NHANES is an open dataset, approved by the NCHS Ethics Review Committee, and all patients/participants provided written informed consent. The hospital ethics committee waived the ethical review of the study. Inclusion criteria for participants were as follows: 1) age ≥20 years; and 2) absence of hearing-related disorders [ear tubes, otoscopic examination abnormalities, embedded earwax, and abnormal binaural tympanic pressure measurements (peak pressure ≤ -150 daPa; compliance ≤0.3 mL)]^19^. Exclusion criteria were: 1) lack of complete hearing data; 2) lack of information on smoking status of exposure factors; and 3) lack of significant covariates [educational level, marital status, income to poverty ratio (PIR), body mass index (BMI), tinnitus, alcohol consumption, hypertension, diabetes, noise exposure, dyslipidemia, and moderate physical activity]. Figure 1 shows a detailed flowchart explaining the selection procedure.

### Audiometry

This study used pure tone audiometry (PTA) as the outcome variable. Trained inspectors determine the air conduction hearing threshold for each ear without hearing aids in the soundproof room of the mobile inspection center. The test was conducted according to the improved Hughson Westlake program, using the automatic testing mode of the audiometer (model AD 226; Interacoustics). Quality assurance and control were establish through daily calibration of equipment and monitoring of environmental noise levels using sound level meters. Participants’ binaural hearing thresholds were evaluated within the frequency range of 0.5–8 kHz. To ensure the accuracy of participants’ responses, each ear underwent two 1 kHz tests^20^. The 0.5, 1, and 2 kHz hearing thresholds were averaged to determine the low-frequency (LF) hearing PTA. The speech-frequency (SF) hearing PTA was obtained by averaging the 0.5, 1, 2, and 4 kHz hearing thresholds. Finally, the high-frequency hearing (HF) PTA was computed by averaging the 4, 6, and 8 kHz hearing thresholds. The audiometrically measured hearing loss is defined as the PTA of 25 dB HL or higher in the better hearing ear as defined by the World Health Organization^21^. Hearing loss is defined in two ways: as a continuous variable and as a categorical variable (PTA <25 vs ≥25 dB HL for the corresponding type of frequency in the better-hearing ear).

### Smoking and covariates

The exposure variable in this study was smoking status, and participants were asked to answer questions about whether they had smoked at least 100 cigarettes in their lifetime. Based on their responses, participants were categorized as non-smokers (<100 cigarettes in their lifetime) and smokers (at least 100 cigarettes in their lifetime). Covariates included sex (female, male), age (younger group aged 20–39 years, middle-aged group aged 40–59 years, older group aged ≥60 years), ethnicity (Mexican American, other Hispanic, non-Hispanic White, non-Hispanic Black, and other races), level of education (<12th grade, high school graduation, or college degree or higher), marital status (married/partner, never married/divorced/separated/widowed), PIR (<1.3, 1.3–3.5, and >3.5), BMI (<25, 25–30, and >30 kg/m^2^), tinnitus (defined based on responses to the question, ‘During the past 12 months, have you been struck by a ringing, ringing, or buzzing sound in your ears or head that lasted for 5 minutes or more? or buzzing sound in your ears or head for 5 minutes or more?’ (based on a ‘yes’ answer to the question, ‘Have you been bothered by a ringing or buzzing sound in your ears or head that lasted 5 minutes or more in the past 12 months?’), alcohol use (e.g. 4 or 5 or more drinks per day), high blood pressure (doctor’s notification of high blood pressure diagnosis on two or more visits), diabetes (based on self-reported diagnosis and/or current use of insulin or other diabetes medication), and noise exposure (including at least any one of the following on the questionnaire: occupational or gun noise or entertainment noise exposure). Noise or recreational noise exposure of at least any one. Occupational noise exposure was assessed by asking the question, ‘Have you ever had a job or jobs that required you to be exposed to loud or noisy noise for 4 or more hours a day, several days a week?’ (Yes/No). Firearm noise exposure is assessed by asking the question, ‘Have you ever used a firearm for any reason?’ (Yes/No). Recreational noise exposure was assessed by the question, ‘Outside of work, are you exposed to noise or music for 10 hours or more per week?’ (Yes/No), dyslipidemia [defined as triglycerides TG ≥150 mg/dL (1.7 mmol/L) or total cholesterol TC ≥200 mg/dL (5.2 mmol/L) or low-density lipoprotein cholesterol LDL-C ≥130 mg/dL (3.4 mmol/L) or high-density lipoprotein cholesterol (HDL-C) ≤40 mg/dL (1.0 mmol/L) or self-reported physician diagnosis or taking cholesterol-lowering medication or taking lipid-lowering medication], moderate work activity (defined as whether the work involves moderate-intensity activity that results in a small increase in respiration or heart rate, such as walking briskly or carrying a light load continuously for at least 10 minutes?)

### Statistical analysis

All analyses used sample weights to account for complex sampling designs in accordance with National Center for Health Statistics guidelines^21^. Continuous variables were expressed as weighted means (95% CI) with p values derived by linear regression; categorical variables were expressed as weighted percentages (95% CI) with p values derived by chi-squared tests. Associations between smoking and hearing loss as continuous and categorical variables (<25 and ≥25 dB HL) were tested using linear and logistic regression analyses, respectively. Three models were developed to assess the β-value and 95% confidence intervals for multiple linear regressions and the odds ratio (OR) and 95% confidence interval (CI) for logistic regressions, respectively, between smoking and hearing thresholds. Model 1 was unadjusted. Model 2 was adjusted for gender, age, race, marital status, and education level. Model 3 was adjusted as for Model 2 plus PIR, BMI, tinnitus, alcohol consumption, hypertension, diabetes, noise exposure, dyslipidemia, and moderate physical activity. In addition to adjusting for these variables, separate subgroup analyses were conducted for gender and age. All analyses were statistically studied using EmpowerStats 6.0 and SPSS V.29.0 (SPSS for Windows, IBM Corp., Armonk, NY), and all data were weighted using WTMEC2YR, SDMVPSU, and SDMVSTRA. Statistical significance was set at two-sided p for trend <0.05.

## RESULTS

### Basic characteristics of the study population

Figure 1 shows the screening process for subjects. A total of 4217 adult subjects (aged ≥20 years) were screened. Of these, 48543 subjects were excluded, including 42867 with missing weighting data, missing hearing data, or hearing-related disorders, and 5676 subjects with no smoking status, covariate data, and age <20 years. The weighted characteristics of the study subjects by smoking status are presented in Supplementary file Table S1, and the chi-squared test showed that there was a statistically significant difference in the distribution of smoking status between the male and female populations (p<0.0001), with 54.33% (n=1143) of males and 45.67% (n=780) of females. There was a statistically significant difference in the distribution of smoking status by age group (p<0.0001), with the highest proportion of smokers in the middle-aged group (40.40%; n=646), followed by the younger group (32.68%; n=600), and the lowest in the older group (26.92%; n=677). Other statistical differences were found in race, marital status, education level, PIR, tinnitus, alcohol consumption, hypertension, diabetes mellitus, noise exposure, dyslipidemia, and moderate physical activity (p<0.05), and no statistical differences were found in BMI (p>0.05). In the categorical variable hearing indicators, we found that smokers had a significantly higher incidence of LFHL (15.62% vs 8.51%), SFHL (23.22% vs 12.98%), and HFHL (53.48% vs 36.95%) than non-smokers (p<0.0001). The same outcome existed for comparisons of hearing metrics using continuous variables (p<0.0001).

### Characteristics of hearing loss in adults

Supplementary file Table S2 characterizes the participants according to the presence of LFHL, SFHL, and HFHL. In our study population, a total of 616 cases were categorized as LFHL, 882 as SFHL, and 1969 as HFHL. We found that the number of smokers was significantly higher than that of non-smokers in all of the populations with LFHL, SFHL, and HFHL (p<0.0001). Non-Hispanic Whites and above high school education level had the highest probability of developing all three types of hearing loss. The older group was most likely to develop LFHL and SFHL, and had a higher risk of developing HFHL. Males were more likely to develop SFHL and HFHL than females, while there was no significant difference in the development of LFHL. Tinnitus, hypertension, diabetes, and high BMI were all more likely to occur in those with LFHL, SFHL, and HFHL (p<0.0001), while there was no difference in PIR and moderate activity (p>0.05). Subjects with noise exposure, alcohol consumption, and dyslipidemia were more likely to develop SFHL and HFHL (p<0.05).

### The relationship between smoking and hearing loss

Table 1 summarizes the results of univariate and multivariable linear and logistic regression analyses that examined the association between smoking and hearing loss. When hearing loss was considered a continuous variable, in Model 1, smoking was associated with LFHL hearing thresholds (β=3.47; 95% CI: 2.79–4.15), SFHL hearing thresholds (β=4.56; 95% CI: 3.81-5.32), and HFHL hearing thresholds (β=8.21; 95% CI: 6.90–9.51) increases were significantly associated (p<0.001). In Model 2, smoking remained significantly associated with increased LFHL, SFHL, and HFHL hearing thresholds (p<0.001). In Model 3, smoking was associated with increased LFHL hearing thresholds (β=1.08; 95% CI: 0.45–1.71, p<0.001), SFHL hearing thresholds (β=1.15; 95% CI: 0.50–1.79, p<0.001), and HFHL hearing threshold (β=1.57; 95% CI: 0.57–2.57, p=0.002) increases were still significantly associated and effect sizes remained large. When hearing loss was considered a categorical variable, in Model 1, smoking was associated with increased LFHL (OR=1.98; 95% CI: 1.67–2.36), SFHL (OR=2.27; 95% CI: 1.95–2.64), and HFHL (OR=2.20; 95% CI: 1.95–2.49) increases were all significantly associated (p<0.001) with large effect sizes. In Models 2 and 3, smoking remained statistically different from SFHL and HFHL (p<0.05) with still large effect sizes; however, there was no statistical difference in LFHL.

**Table 1:** Linear and logistic regression analysis of the relationship between smoking and hearing loss among all participants in different models, NHANES 2005–2012 and 2015–2018

*Variables*	*Model 1*	*Model 2*	*Model 3*
*Continuous*	β (95% CI) p*	β (95% CI) p*	β (95% CI) p*
LFHL	3.47 (2.79–4.15) <0.001	1.28 (0.66–1.89) <0.001	1.08 (0.45–1.71) <0.001
SFHL	4.56 (3.81–5.32) <0.001	1.50 (0.86–2.14) <0.001	1.15 (0.50–1.79) <0.001
HFHL	8.21 (6.90–9.51) <0.001	2.23 (1.23–3.23) <0.001	1.57 (0.57–2.57) 0.002
***Categorical***	OR (95% CI) p*	AOR (95% CI) p*	AOR (95% CI) p*
LFHL	1.98 (1.67–2.36) <0.001	1.22 (0.99–1.49) 0.058	1.19 (0.97–1.47) 0.102
LFHL	2.27 (1.95–2.64) <0.001	1.33 (1.10–1.60) 0.003	1.27 (1.04–1.54) 0.017
HFHL	2.20 (1.95–2.49) <0.001	1.38 (1.17–1.63) <0.001	1.26 (1.06–1.50) 0.008

Model 1: crude model with no covariates. AOR: adjusted odds ratio. Model 2: adjusted for gender, age, race, education level, and marital status. Model 3: adjusted as for Model 2 plus drinking, PIR, tinnitus, noise exposure, BMI, hypertension, dyslipidemia, diabetes, and moderate physical activity.

* p for trend.

### Gender and age subgroup analysis

We analyzed subgroups for gender (Table 2) and age (Table 3), respectively. Table 2 examines the association between smoking and different frequencies of hearing loss in male and female subjects, respectively.

**Table 2:** Linear and logistic regression analysis of the relationship between smoking and hearing loss among participants, by gender, in different models NHANES 2005–2012 and 2015–2018

*Variables*	*Model 1*	*Model 2*	*Model 3*
*Continuous*	β (95% CI) p*	β (95% CI) p*	β (95% CI) p*
LFHL (Female)	2.71 (1.71–3.72) <0.001	0.86 (-0.04–1.76) 0.062	0.70 (-0.23–1.62) 0.141
LFHL (Male)	4.24 (3.32–5.17) <0.001	1.65 (0.80–2.49) <0.001	1.52 (0.66–2.38) <0.001
SFHL (Female)	3.08 (2.01–4.14) <0.001	0.87 (-0.04–1.79) 0.060	0.63 (-0.30–1.56) 0.185
SFHL (Male)	5.63 (4.56–6.70) <0.001	1.95 (1.05–2.84) <0.001	1.62 (0.72–2.52) <0.001
HFHL (Female)	4.99 (3.35–6.64) <0.001	1.11 (-0.16–2.37) 0.087	0.62 (-0.66–1.90) 0.343
HFHL (Male)	10.20 (8.21–12.19) <0.001	2.85 (1.33–4.37) <0.001	2.19 (0.69–3.70) 0.004
***Categorical***	OR (95% CI) p*	AOR (95% CI) p*	AOR (95% CI) p*
LFHL (Female)	1.59 (1.23–2.06) <0.001	1.08 (0.80–1.44) 0.616	1.08 (0.80–1.46) 0.626
LFHL (Male)	2.36 (1.84–3.03) <0.001	1.39 (1.05–1.85) 0.022	1.35 (1.01–1.81) 0.045
SFHL (Female)	1.72 (1.35–2.18) <0.001	1.16 (0.88–1.54) 0.301	1.19 (0.88–1.59) 0.258
SFHL (Male)	2.50 (2.03–3.06) <0.001	1.51 (1.17–1.94) <0.001	1.39 (1.07–1.81) 0.013
HFHL (Female)	1.82 (1.52–2.18) <0.001	1.19 (0.93–1.53) 0.169	1.09 (0.84–1.41) 0.537
HFHL (Male)	2.40 (2.01–2.84) <0.001	1.56 (1.24–1.95) <0.001	1.43 (1.13–1.81) 0.003

Model 1: crude model with no covariates. AOR: adjusted odds ratio. Model 2: adjusted for gender, age, race, education level, and marital status. Model 3: adjusted as for Model 2 plus drinking, PIR, tinnitus, noise exposure, BMI, hypertension, dyslipidemia, diabetes, and moderate physical activity.

* p for trend.

**Table 3:** Linear and logistic regression analysis of the relationship between smoking and hearing loss among participants, by age (years), in different models, NHANES 2005–2012 and 2015–2018

*Variables*	*Model 1*	*Model 2*	*Model 3*
*Continuous*	β (95% CI) p*	β (95% CI) p*	β (95% CI) p*
LFHL (20–39)	1.09 (0.39–1.78) 0.002	0.68 (-0.02–1.38) 0.056	0.28 (-0.44–0.99) 0.447
LFHL (40–59)	2.75 (1.74–3.76) <0.001	2.38 (1.33–3.43) <0.001	2.19 (1.12–3.25) <0.001
LFHL (≥60)	1.41 (-0.23–3.05) 0.093	0.62 (-1.02–2.25) 0.460	0.25 (-1.41–1.90) 0.770
SFHL (20–39)	1.52 (0.80–2.25) <0.001	0.95 (0.23–1.67) 0.010	0.42 (-0.31–1.15) 0.262
SFHL (40–59)	3.17 (2.08–4.27) <0.001	2.27 (1.16–3.39) <0.001	1.89 (0.79–3.00) <0.001
SFHL (≥60)	2.60 (0.90–4.31) 0.003	0.96 (-0.70–2.62) 0.256	0.55 (-1.12–2.22) 0.519
HFHL (20–39)	1.81 (0.70–2.93) 0.001	1.03 (-0.07–2.13) 0.067	0.46 (-0.66–1.59) 0.420
HFHL (40–59)	4.55 (2.63–6.47) <0.001	2.54 (0.65–4.43) 0.009	1.59 (-0.24–3.41) 0.088
HFHL (≥60)	6.16 (3.67–8.65) <0.001	2.60 (0.26–4.93) <0.029	1.83 (-0.51–4.16) 0.126
***Categorical***	OR (95% CI) p*	AOR (95% CI) p*	AOR (95% CI) p*
LFHL (20–39)	1.05 (0.51–2.16) 0.895	0.92 (0.43–1.97) 0.834	0.74 (0.32–1.71) 0.478
LFHL (40–59)	2.37 (1.61–3.49) <0.001	2.09 (1.40–3.12) <0.001	1.97 (1.30–2.980) 0.001
LFHL (≥60)	1.16 (0.92–1.48) 0.218	1.04 (0.80–1.34) 0.788	1.04 (0.80–1.35) 0.799
SFHL (20–39)	0.90 (0.48–1.70) 0.749	0.70 (0.36–1.36) 0.296	0.50 (0.24–1.05) 0.067
SFHL (40–59)	2.30 (1.70–3.12) <0.001	1.83 (1.33–2.52) <0.001	1.68 (1.21–2.35) 0.002
SFHL (≥60)	1.54 (1.22–1.94) <0.001	1.25 (0.97–1.62) 0.092	1.23 (0.94–1.61) 0.128
HFHL (20–39)	1.55 (1.16–2.07) 0.003	1.24 (0.91–1.68) 0.174	1.11 (0.80–1.54) 0.542
HFHL (40–59)	1.83 (1.48–2.27) <0.001	1.41 (1.13–1.77) 0.003	1.30 (1.02–1.64) 0.034
HFHL (≥60)	1.93 (1.30–2.85) 0.001	1.55 (1.02–2.36) 0.041	1.35 (0.87–2.10) 0.179

Model 1: crude model with no covariates. AOR: adjusted odds ratio. Model 2: adjusted for gender, age, race, education level, and marital status. Model 3: adjusted as for Model 2 plus drinking, PIR, tinnitus, noise exposure, BMI, hypertension, dyslipidemia, diabetes, and moderate physical activity.

* p for trend.

Among males, hearing loss, continuous variables showed statistically significant differences (p<0.05) in Models 1, 2 and 3 for LFHL (β=4.24; 95% CI: 3.32–5.17; β=1.65; 95% CI: 0.80–2.49; β=1.52; 95% CI: 0.66–2.38), SFHL (β=5.63; 95% CI: 4.56–6.70; β=1.95; 95% CI: 1.05–2.84; β=1.62; 95% CI: 0.72– 2.52) and HFHL (β=10.20; 95% CI: 8.21–12.19; β=2.85; 95% CI: 1.33–4.37; β=2.19; 95% CI: 0.69– 3.70) between smokers and non-smokers. In male hearing loss, between smokers and non-smokers, categorical variables also showed statistically significant differences (p<0.05) in Models 1, 2 and 3 for LFHL (OR=2.36; 95% CI: 1.84–3.03; AOR=1.39; 95% CI: 1.05–1.85; AOR=1.35; 95% CI: 1.01–1.81), SFHL (OR=2.50; 95% CI: 2.03–3.06; AOR=1.51; 95% CI: 1.17–1.94; AOR=1.39; 95% CI: 1.07–1.81) and HFHL (OR=2.39; 95% CI: 2.01–2.84; AOR=1.56; 95% CI: 1.24–1.95; AOR=1.43; 95% CI: 1.13–0.812). For female subjects, statistically significant differences with large effect values were shown in Model 1 for both linear and logistic regression, but in Models 2 and 3, there were no statistically significant differences (p>0.05). Table 3 analyzes smoking and hearing loss for the three age subgroups of adult subjects. In the middle-aged group, smoking and the three types of hearing loss in the logistic regression showed statistically significant differences (p<0.05) in Models 1, 2 and 3. In linear regression, the middle-aged group did not show statistical differences only in Model 3 for HFHL and in all three models for LFHL, SFHL, and Models 1 and 2 for HFHL. In logistic regression, there were no statistical differences in the younger group in all three models of LFHL and SFHL, and in Models 2 and 3 of HFHL. In the older group of LFHL subjects, there was no statistical difference in any of the three models of linear and logistic regression (p>0.05). In the older group, both SFHL and HFHL showed statistical differences in Model 1 in linear and logistic regression but none in Model 3. More details can be found in the Supplementary file.

## DISCUSSION

In our cross-sectional study of 4217 adult subjects, there was a significant difference in the higher proportion of males compared with females who smoked cigarettes, consistent with the literature^1^. Recent studies have reported that hearing impairment is more common in patients with hypertension, diabetes mellitus, and tinnitus compared to healthy subjects^8,9,22^, which is in line with the findings of our study; LFHL, SFHL, and HFHL were all equally more susceptible to the above risks in all three groups. In addition, we also found that all three of these hearing losses were also more prevalent in subjects with high BMI, which may be related to the fact that high BMI is often comorbid with hypertension and diabetes. This is consistent with studies reporting a strong positive correlation between obesity, overweight, and hearing loss in the literature^7,9,23^. In the literature^1,16,24^, subjects with other risk factors, noise exposure, alcohol consumption, and hyperlipidemia were also found to be more prone to SFHL and HFHL, but there was no significant correlation between LFHL and these factors.

From both continuous and categorical variables of hearing, the present study confirms that smokers develop LFHL, SFHL, and HFHL significantly more than non-smokers; and in the populations with LFHL, SFHL, and HFHL, the number of smokers was also found to be considerably higher than that of non-smokers in subjects with all three types of hearing loss. Several mechanisms are proposed that may underlie the relationship between smoking and hearing loss. Cigarette smoking has been associated with increased production of reactive oxygen species and increased oxidative stress^25,26^, elevated systemic markers of inflammation^27^ and damage to the outer hair cells of the cochlea^28^. Smoking-associated redox system damage is associated with cochlear hypoxia, cellular damage, and mechanisms by which reactive substances damage outer hair cells^12,25,26^. Components of cigarette smoke, such as toluene, benzene, and carbon monoxide, have also been associated with hearing loss^29,30^. Cigarette smoking may also impair vascular endothelial function, increase the risk of atherosclerosis, increase blood viscosity, and impair oxygen delivery to the cochlea^31,32^. Nicotine in tobacco may cause vasoconstriction, impair tissue perfusion, and lead to cellular dysfunction^32,33^. We also adjusted the model for covariates and found that smoking was strongly associated with both SFHL and HFHL, while the correlation with LFHL was not significant. The stronger association between smoking and HF hearing loss than LF hearing loss may be due to the direct ototoxic effects of tobacco on cochlear outer hair cell function^33^ or the greater negative impact on HF hearing loss by impairing the auditory system through an increase in carboxyhemoglobin and a decrease in blood flow to the cochlea^34,35^.

Previous studies have mostly explored the relationship between smoking and hearing loss by adjusting for age rather than analyzing the relationship in specific age subgroups after controlling for other covariates^1^. Therefore, we hypothesized that the effect of smoking on hearing loss may vary by gender and age. Thus, in this study, we investigated the relationship between smoking status and hearing loss by adjusting for covariates to create three models and analyzing continuous and categorical variables of hearing loss separately, especially in stratified gender and age groups. We found that among men, hearing loss, both continuous and categorical variables, showed statistically significant differences in Model 1, Model 2, and Model 3 for LFHL, SFHL, and HFHL, with large effect values. Thus, we can see that male smokers have a significant relationship with all three types of hearing loss. A study^13^ of workers at Dongfeng Motor Company in China and another study^1^ of the general population in Zhejiang Province, China, both found that smoking was not significantly associated with hearing loss in the female population. They attributed this to the fact that the prevalence of smoking in females (2.6% and 2.0%, respectively) was much lower than that of males, resulting in limited statistical efficacy. In the present study, the smoking prevalence was significantly higher among adult females in the United States (n=780; 45.67%) than among adult females in China; however, we also did not find a significant relationship between smoking and the three types of hearing loss among females. However, a longitudinal study of White women found that both past and present smoking were associated with a higher risk of moderate or severe hearing loss^12^. The relationship between smoking and hearing loss in the female population is still controversial and needs to be explored in further studies with larger populations and samples. Female smokers are often thought to smoke less on average than male smokers, and there may be differences in sensitivity to smoking damage by gender, which need to be further explored and researched.

From the three age subgroups, we found that smoking in the middle-aged group was significantly associated with LFHL, SFHL, and HFHL, whereas smoking in the middle-aged group was reported in the literature to be substantially associated with SFHL and HFHL only. However, in the younger group, we found that smoking was not associated with LFHL, SFHL, and HFHL, which is similar to the literature reporting that smoking was not significantly associated with SFHL and HFHL in young men^1^. The risk of hearing loss increased with age, with young men having the lowest prevalence of hearing loss. The damage caused by tobacco to human health does not become apparent until years or even decades after use^36^. Among young men, alcohol consumption, noise, hypertension, and diabetes were significantly associated with hearing loss, but smoking was not. We believe that the negative effect of smoking on hearing loss is not significant in young people. In the middle-aged group, the negative effects of smoking have been accumulating for many years, and the effects on hearing loss have become apparent, along with other risk factors such as noise, hypertension, and diabetes. In the older age group, smoking was also not significantly associated with LFHL, SFHL, and HFHL, similar to the lack of a significant relationship between smoking and LFHL and HFHL reported in the literature. Wang et al.^13^ found that the correlation between current smoking and speech frequency hearing loss was not significant in individuals aged >70 years. The negative impact of smoking on hearing loss is likely to be offset by aging-related hearing loss due to physiological dysfunction or chronic diseases such as hypertension and hyperlipidemia.

### Strengths and limitations

This study has several strengths. First, it is the first large-scale, population-based investigation using NHANSES and fills a gap in the lack of research on the relationship between smoking and different types of hearing loss, particularly across gender and age groups. We analyzed the relationship between smoking and hearing loss in stratified age subgroups, rather than adjusting for age, to provide a more detailed picture of the impact of smoking on hearing loss. Second, we chose nine frequency bands of pure tone threshold audiometry for hearing separately for continuous and categorical variables rather than self-reported hearing loss or narrow frequency bands, which allowed a more precise assessment of hearing loss. Finally, the questionnaire used included a broad set of covariates, excluding many potential confounders. However, there are some limitations to this study. First, the cross-sectional design limited the evidence for causal inferences. Second, the questionnaire was used to collect information about smoking habits and disease history based on participants’ self-reports, and there was no secondary source to confirm the accuracy of responses. Third, more than 10 variables were included in this study, and regression analyses were performed under the assumption that all of these included covariates were independent of each other. Fourth, additional restrictions include residual confounding factors, which have limited applicability to other countries.

## CONCLUSIONS

The relationship between smoking and hearing loss varied by sex and age. Smoking was significantly associated with low-frequency, speech-frequency, and high-frequency hearing loss in males but not in females. Smoking was significantly associated with LFHL, SFHL, and HFHL in the middle-aged group, but not in the younger and older age groups.

## Supplementary Material



## Data Availability

Publicly available datasets were analyzed in this study. The data supporting this research are available from the following source: https://wwwn.cdc.gov/nchs/nhanes/.
