# Supplementary material for: Association between cigarette smoking and hearing loss: A cross-sectional study from the NHANES database
Source: Tob Induc Dis. 2025 Sep 17;23:10.18332/tid/208812. doi: 10.18332/tid/208812 (PMC12442883; doi:10.18332/tid/208812)
Supplement: Supplementary file 1 [file TID-23-133-s1.pdf]

Supplementary file

Table S1. Weighted characteristics of research subjects by adult smoking status in NHANES from 2005 to 2018 (n=4217) (2005-2012 and 2015-2018)

	**Non-smoking**	**smoking**	**p**
Gender			<0.0001
Female	(1261) 54.67 (52.30 ,57.03)	(780) 45.67 (42.69 ,48.68)	
Male	(1033) 45.33 (42.97 ,47.70)	(1143) 54.33 (51.32 ,57.31)	
Age (years)			<0.0001
Young (20-39)	(1049) 46.51 (42.32 ,50.76)	(600) 32.68 (29.29 ,36.26)	
Middle-aged (40-59)	(755) 36.98 (33.31 ,40.82)	(646) 40.40 (36.47 ,44.46)	
Older (≥60)	(490) 16.50 (14.13 ,19.19)	(677) 26.92 (23.91 ,30.16)	
Race			<0.0001
Mexican american	(311) 8.49 (5.84 ,12.18)	(230) 6.74 (4.94 ,9.14)	
Other hispanic	(251) 6.29 (4.51 ,8.72)	(171) 4.70 (3.08 ,7.09)	
Non-hispanic white	(857) 67.72 (62.17 ,72.80)	(957) 75.34 (70.59 ,79.54)	
Non-hispanic black	(525) 10.36 (7.82 ,13.62)	(374) 7.71 (5.81 ,10.15)	
Other races	(350) 7.14 (5.67 ,8.95)	(191) 5.52 (4.26 ,7.11)	
Educational level			<0.0001
Less than high school	(329) 8.71 (6.79 ,11.11)	(442) 15.67 (12.78 ,19.07)	
High school or GED	(383) 14.07 (11.85 ,16.63)	(467) 23.59 (20.51 ,26.98)	
Above high school	(1582) 77.22 (73.06 ,80.91)	(1014) 60.74 (55.83 ,65.45)	
Marital status			0.1338
Married/living with partner	(1417) 66.38 (62.33 ,70.20)	(1144) 63.13 (59.59 ,66.52)	
Never married/divorced/ separated/widowed	(877) 33.62 (29.80 ,37.67)	(779) 36.87 (33.48 ,40.41)	
Alcohol			<0.0001
No	(2114) 93.05 (91.04 ,94.64)	(1435) 74.69 (71.87 ,77.32)	
Yes	(180) 6.95 (5.36 ,8.96)	(488) 25.31 (22.68 ,28.13)	
PIR			<0.0001
>3,5	(883) 50.37 (45.05 ,55.69)	(535) 39.89 (34.59 ,45.43)	
1.3-3.5	(846) 33.42 (29.70 ,37.35)	(776) 37.66 (33.73 ,41.77)	
1.3	(565) 16.21 (13.32 ,19.58)	(612) 22.45 (19.57 ,25.62)	
Tinnitus			0.0010
No	(1989) 85.39 (82.72 ,87.71)	(1544) 80.20 (77.44 ,82.70)	
Yes	(305) 14.61 (12.29 ,17.28)	(379) 19.80 (17.30 ,22.56)	
Noise exposure			<0.0001
No	(1152) 43.13 (39.66 ,46.67)	(620) 29.14 (26.02 ,32.47)	
Yes	(1142) 56.87 (53.33 ,60.34)	(1303) 70.86 (67.53 ,73.98)	
BMI (kg/m2)			0.1148
<25	(697) 30.51 (26.55 ,34.79)	(521) 28.18 (24.89 ,31.72)	
25-30	(753) 34.46 (31.17 ,37.90)	(651) 31.78 (28.72 ,35.02)	
>30	(844) 35.03 (31.61 ,38.61)	(751) 40.04 (36.64 ,43.53)	

Hypertension				0.0001
	No	(1542) 69.73 (66.92 ,72.41)	(1144) 63.00 (60.57 ,65.37)	
	Yes	(752) 30.27 (27.59 ,33.08)	(779) 37.00 (34.63 ,39.43)	
Abnormal blood lipids				<0.0001
	No	(1050) 44.44 (41.84 ,47.06)	(748) 36.54 (33.78 ,39.40)	
	Yes	(1244) 55.56 (52.94 ,58.16)	(1175) 63.46 (60.60 ,66.22)	
Diabetes				0.0107
	No	(2082) 93.23 (91.50 ,94.63)	(1655) 90.21 (88.41 ,91.75)	
	Yes	(212) 6.77 (5.37 ,8.50)	(268) 9.79 (8.25 ,11.59)	
Moderate activity				0.0002
	No	(1433) 58.42 (55.58 ,61.20)	(1115) 53.09 (49.83 ,56.32)	
	Yes	(861) 41.58 (38.80 ,44.42)	(808) 46.91 (43.68 ,50.17)	
LFHL				<0.0001
	No	(2048) 91.49 (89.72 ,92.98)	(1553) 84.38 (81.91 ,86.56)	
	Yes	(246) 8.51 (7.02 ,10.28)	(370) 15.62 (13.44 ,18.09)	
SFHL				<0.0001
	No	(1955) 87.02 (84.72 ,89.02)	(1380) 76.78 (74.08 ,79.28)	
	Yes	(339) 12.98 (10.98 ,15.28)	(543) 23.22 (20.72 ,25.92)	
HFHL				<0.0001
	No	(1426) 63.05 (58.70 ,67.19)	(822) 46.52 (42.47 ,50.63)	
	Yes	(868) 36.95 (32.81 ,41.30)	(1101) 53.48 (49.37 ,57.53)	
LFHL(continuous)		(2294) 11.10 (10.41 ,11.78)	(1923) 14.56 (13.75 ,15.38)	<0.0001
SFHL(continuous)		(2294) 13.10 (12.26 ,13.94)	(1923) 17.66 (16.71 ,18.62)	<0.0001
HFHL(continuous)		(2294) 25.34 (23.60 ,27.08)	(1923) 33.55 (31.84 ,35.25)	<0.0001

P values from $\chi 2$ test or Wilcoxon rank-sum test (categorical categories) and Student's t-tests (continuous covariates). *P < 0.05, **P < 0.01, ***P < 0.001. Data in the table:For continuous variables: (N-observe, N-represent) survey-weighted mean (95% CI) , P-value was by survey-weighted linear regression (svyglm); For categorical variables: (N-observe, N-represent) survey-weighted percentage (95% CI) , P-value was by survey-weighted Chi-square test (svytable).

Table S2. Baseline characteristics of study participants in NHANES according to hearing loss (2005-2012 and 2015-2018)

Characteristics	Non-LFHL (N = 3601)	LFHL (N = 616)	P-value	Non-SFHL (N = 3335)	SFHL (N = 882)	P-value	Non-HFHL (N = 2248)	HFHL (N = 1969)	P
Gender			0.5517			<0.0001			<0.0001
Female	50.79 (49.25 , 52.34)	49.15 (43.41 ,54.92)		52.88 (51.09 ,54.67)	39.94 (35.32 ,44.74)		55.10 (52.89 , 57.29)	44.98 (42.39 , 47.59)	
Male	49.21 (47.66 , 50.75)	50.85 (45.08 ,56.59)		47.12 (45.33 ,48.91)	60.06 (55.26 ,64.68)		44.90 (42.71 , 47.11)	55.02 (52.41 , 57.61)	
Age (years)			<0.0001			<0.0001			<0.0001
Young (20-39)	44.78 (40.95 , 48.68)	6.17 (4.08 , 9.22)		47.81 (44.03 ,51.61)	4.92 (3.44 , 7.00)		63.47 (59.57 , 67.19)	11.22 (9.40 ,13.34)	
Middle-aged (40-59)	40.06 (36.70 , 43.53)	26.98 (20.42 ,34.73)		39.39 (36.03 ,42.85)	34.52 (29.38 ,40.04)		32.89 (29.17 , 36.83)	45.59 (41.52 , 49.72)	
Older (⩾60)	15.15 (13.49 , 16.98)	66.85 (59.27 ,73.65)		12.81 (11.23 ,14.57)	60.56 (55.24 ,65.64)		3.65 (2.50 ,5.29)	43.19 (39.30 , 47.17)	
Race			<0.0001			<0.0001			<0.0001
Mexican american	8.14 (5.82 ,11.28)	4.35 (2.81 , 6.67)		8.41 (5.99 , 11.68)	4.37 (2.95 , 6.43)		9.40 (6.54 ,13.34)	5.57 (3.91 ,7.89)	
Other hispanic	5.85 (4.16 ,8.16)	3.50 (1.86 , 6.49)		5.99 (4.26 , 8.36)	3.62 (2.12 , 6.12)		6.77 (4.77 ,9.52)	4.07 (2.74 ,6.01)	
Non-hispanic white	69.90 (64.65 , 74.67)	80.69 (75.85 ,84.75)		69.00 (63.54 ,73.98)	81.28 (76.76 ,85.09)		65.55 (59.80 , 70.89)	78.18 (73.91 , 81.92)	
Non-hispanic black	9.50 (7.16 ,12.50)	6.65 (4.85 , 9.06)		9.95 (7.49 , 13.11)	5.46 (4.09 , 7.25)		10.93 (8.18 ,14.46)	6.95 (5.28 ,9.12)	
Other races	6.62 (5.28 ,8	4.81 (3.20 ,		6.65 (5.29 ,	5.27 (3.39 ,		7.35 (5.75 ,9	5.22 (4.07 ,6	

	.26)	7.18)		8.33)	8.09)		.35)	.68)	
Educational level			<0.0001			<0.0001			<0.0001
Less than high school	10.72 (8.72 ,13.10)	20.42 (16.34 ,25.20)		10.17 (8.27 , 12.45)	19.74 (15.76 ,24.44)		8.43 (6.36 ,11.09)	16.14 (13.44 , 19.26)	
High school or GED	17.88 (15.79 , 20.19)	22.08 (17.81 ,27.04)		17.31 (15.16 ,19.71)	23.35 (18.90 ,28.48)		15.33 (12.76 , 18.32)	22.18 (19.37 , 25.27)	
Above high school	71.40 (67.46 , 75.03)	57.50 (50.58 ,64.14)		72.52 (68.65 ,76.07)	56.90 (49.85 ,63.69)		76.24 (71.49 , 80.41)	61.68 (56.96 , 66.18)	
Marital status			0.9250			0.3506			0.0238
Married/living with partner	64.94 (61.62 , 68.12)	64.66 (59.02 ,69.92)		64.43 (60.92 ,67.79)	67.12 (62.02 ,71.85)		62.01 (58.06 , 65.80)	68.53 (63.90 , 72.83)	
Never married/divorced/ separated/widowed	35.06 (31.88 , 38.38)	35.34 (30.08 ,40.98)		35.57 (32.21 ,39.08)	32.88 (28.15 ,37.98)		37.99 (34.20 , 41.94)	31.47 (27.17 , 36.10)	
Alcohol			0.0753			0.0140			0.0002
No	85.24 (83.24 , 87.04)	81.03 (74.49 ,86.20)		85.60 (83.63 ,87.36)	80.77 (75.41 ,85.20)		87.47 (84.62 , 89.85)	81.34 (78.65 , 83.76)	
Yes	14.76 (12.96 , 16.76)	18.97 (13.80 ,25.51)		14.40 (12.64 ,16.37)	19.23 (14.80 ,24.59)		12.53 (10.15 , 15.38)	18.66 (16.24 , 21.35)	
Smoking			<0.0001			<0.0001			<0.0001
No	56.77 (54.25 , 59.24)	39.75 (34.29 ,45.47)		57.85 (55.39 ,60.26)	40.37 (35.88 ,45.02)		62.13 (59.08 , 65.09)	45.56 (42.73 , 48.41)	
Yes	43.23 (40.76 , 45.75)	60.25 (54.53 ,65.71)		42.15 (39.74 ,44.61)	59.63 (54.98 ,64.12)		37.87 (34.91 , 40.92)	54.44 (51.59 , 57.27)	
PIR			0.0683			0.1926			0.4745
>3,5	46.26 (41.54 , 51.05)	40.90 (33.29 ,48.98)		46.42 (41.66 ,51.25)	41.93 (34.96 ,49.23)		44.25 (39.19 , 49.44)	47.35 (41.24 , 53.54)	

1.3-3.5	34.50 (31.23 , 37.92)	41.63 (35.27 ,48.28)		34.34 (30.97 ,37.87)	40.02 (34.04 ,46.32)		35.75 (32.10 , 39.57)	34.83 (30.74 , 39.14)	
1.3	19.24 (16.61 , 22.17)	17.46 (13.96 ,21.63)		19.24 (16.38 ,22.46)	18.05 (14.39 ,22.39)		20.00 (16.56 , 23.95)	17.82 (14.66 , 21.49)	
Tinnitus			<0.0001			<0.0001			<0.0001
No	85.25 (82.90 , 87.32)	66.49 (60.68 ,71.83)		86.93 (84.80 ,88.80)	64.88 (60.59 ,68.95)		91.11 (89.40 , 92.56)	72.96 (69.55 , 76.12)	
Yes	14.75 (12.68 , 17.10)	33.51 (28.17 ,39.32)		13.07 (11.20 ,15.20)	35.12 (31.05 ,39.41)		8.89 (7.44 ,10.60)	27.04 (23.88 , 30.45)	
Noise exposure			0.3538			0.0003			0.0012
No	37.10 (34.02 , 40.28)	34.60 (29.75 ,39.78)		38.40 (35.37 ,41.52)	29.34 (24.83 ,34.30)		39.19 (35.89 , 42.59)	33.81 (30.58 , 37.21)	
Yes	62.90 (59.72 , 65.98)	65.40 (60.22 ,70.25)		61.60 (58.48 ,64.63)	70.66 (65.70 ,75.17)		60.81 (57.41 , 64.11)	66.19 (62.79 , 69.42)	
BMI (kg/m2)			0.0116			0.0007			<0.0001
<25	30.41 (27.26 , 33.75)	22.33 (18.00 ,27.36)		31.10 (27.83 ,34.56)	21.80 (18.35 ,25.69)		34.47 (30.81 , 38.32)	23.19 (20.47 , 26.16)	
25-30	32.97 (30.67 , 35.35)	35.37 (29.51 ,41.70)		33.04 (30.54 ,35.64)	34.22 (29.15 ,39.68)		31.40 (28.88 , 34.04)	35.56 (32.23 , 39.03)	
>30	36.63 (33.87 , 39.47)	42.30 (37.38 ,47.37)		35.86 (33.03 ,38.80)	43.98 (38.83 ,49.27)		34.13 (30.63 , 37.82)	41.25 (38.37 , 44.18)	
Hypertension			<0.0001			<0.0001			<0.0001
No	68.20 (65.89 , 70.42)	55.30 (51.20 ,59.33)		69.52 (67.07 ,71.85)	53.47 (49.22 ,57.66)		74.50 (71.84 , 76.98)	56.92 (54.15 , 59.65)	
Yes	31.80 (29.58 , 34.11)	44.70 (40.67 ,48.80)		30.48 (28.15 ,32.93)	46.53 (42.34 ,50.78)		25.50 (23.02 , 28.16)	43.08 (40.35 , 45.85)	
Abnormal blood lipids			0.2667			<0.0001			<0.0001
No	41.20 (39.15 ,	38.34 (33.56		42.64 (40.56	32.57 (28.71		48.99 (46.28 ,	30.71 (28.73 ,	

		43.28)	,43.36)		,44.75)	,36.69)		51.71)	32.75)	
	Yes	58.80 (56.72 , 60.85)	61.66 (56.64 ,66.44)		57.36 (55.25 ,59.44)	67.43 (63.31 ,71.29)		51.01 (48.29 , 53.72)	69.29 (67.25 , 71.27)	
Diabetes				<0.0001			<0.0001			<0.0001
	No	93.01 (91.71 , 94.12)	83.25 (78.60 ,87.06)		93.83 (92.46 ,94.96)	82.68 (78.11 ,86.46)		96.06 (94.68 , 97.10)	86.61 (84.29 , 88.64)	
	Yes	6.99 (5.88 ,8 .29)	16.75 (12.94 ,21.40)		6.17 (5.04 , 7.54)	17.32 (13.54 ,21.89)		3.94 (2.90 ,5 .32)	13.39 (11.36 , 15.71)	
Moderate activity				0.1119			0.2017			0.4418
	No	55.49 (52.67 , 58.29)	59.85 (54.46 ,65.02)		55.39 (52.44 ,58.31)	58.86 (53.77 ,63.77)		55.34 (51.93 , 58.69)	56.84 (53.48 , 60.14)	
	Yes	44.51 (41.71 , 47.33)	40.15 (34.98 ,45.54)		44.61 (41.69 ,47.56)	41.14 (36.23 ,46.23)		44.66 (41.31 , 48.07)	43.16 (39.86 , 46.52)	

For categorical variables: survey-weighted percentage (95%CI), P was by survey-weighted Chi-square test (svytable)

© 2025 Huang H. et al.
